# Potential DPP IV Inhibitory Peptides from Dry-Cured Pork Loins after Hydrolysis: An In Vitro and In Silico Study

**DOI:** 10.3390/cimb43030095

**Published:** 2021-09-27

**Authors:** Paulina Kęska, Joanna Stadnik

**Affiliations:** Department of Animal Food Technology, Faculty of Food Science and Biotechnology, University of Life Sciences in Lublin, Skromna 8, 20-704 Lublin, Poland; paulina.keska@up.lublin.pl

**Keywords:** DPP-IV, peptides, dry-cured meat, spectrometric analysis

## Abstract

Peptidyl peptidase IV (DPP-IV) is a pharmacotherapeutic target in type 2 diabetes, and inhibitors of this enzyme are an important class of drugs for the treatment of type 2 diabetes. In the present study, peptides (<7 kDa) isolated from dry-cured pork loins after pepsin and pancreatin hydrolysis were identified by mass spectrometry and tested as potential inhibitors of DPP-IV by the in silico method. Two peptides, namely WTIAVPGPPHS from myomesin (water-soluble fraction, A = 0.9091) and FKRPPL from troponin (salt-soluble fraction, A = 0.8333), were selected as the most promising inhibitors of DPP-IV. Both peptides were subjected to ADMET analysis. Fragments of these peptides showed promising drug-likeness properties as well as favorable absorption, distribution, metabolism, excretion, and toxicity functions, suggesting that they are novel leads in the development of DPP-IV inhibitors from food.

## 1. Introduction

Recent advances in analytical techniques have enabled rapid and more accurate detection and identification of proteins and peptides. The application of peptidomic approaches, including advanced mass spectrometry methodologies and bioinformatics tools, is fundamental to the discovery and identification of peptides in food. Unique peptide isolated from different species and those from carcass or specific food types can survive the effect of time, heat treatment, and other technological processes of animal products, such as fermentation or aging. For example, differences in the peptide profile were observed in different types of hams probably due to varying curing times [1]. These peptides could potentially be used to evaluate the best time and conditions for processing meat to achieve the desired flavor and texture. Scientists have also discovered other favorable properties of meat peptides, including their biological effect on the human body. Currently, there is a strong trend in scientific research to discover, validate, and demonstrate the added value of food and its nutritional effect [2]. Recently, peptide extracts from dry-cured or fermented meat products have been used in peptidomics research because of their angiotensin I-converting enzyme (ACE-I) inhibitory activity and antioxidant effects [1,3,4,5,6]. The influence of aging time on the content of peptides in protein extracts and on their biological activity as an ACE-I inhibitor and antioxidants was determined earlier [4,5]. A new class of biopeptides, namely dipeptidyl peptidase IV (DPP-IV) inhibitors, has recently gained interest from researchers. DPP-IV is involved in the metabolic pathways responsible for glucose metabolism. DPP-IV hydrolyzes incretins such as glucose insulinotropic peptide (GIP) or glucagon-like peptide-1 (GLP-1) and causes their inactivation. Therefore, inhibiting DPP-IV may prevent the degradation of these hormones. This allows them to maintain an adequate level of glucose in the plasma by maintaining insulinotropic activity in the postprandial phase. Galleo et al. [7] proved that DPP-IV inhibitor peptides (KA, AAATP, AA, GP, PL, and carnosine as well as AAAAG, ALGGA, and LVSGM peptides) can be present in water-soluble dried ham extract. The high potential of food products to produce peptides with DPP-IV inhibitory activity has also been demonstrated [8]. However, the identification of novel peptides that inhibit the action of the meat protein DPP-IV by using a proteomic approach remains to be conducted.

In the present study, the differences in peptides due to the passage of time during product maturation were analyzed by mass spectrometry. The aging-dependent peptides of proteins and polypeptides can serve as unique indicators that may help to choose the ripening time of the loin and to optimize the production parameters in order to obtain a product with better performance characteristics. Based on the sequences of the peptides, proteins were identified as their source of origin, and their functional interaction was then analyzed to determine the relationship between the functions of proteins that are a stable source of DPP-IV inhibitors.

Synthetic bioactive peptides have long been used in therapeutic applications as drug components contributing to the treatment of obesity, diabetes, osteoporosis, and cardiovascular, gastrointestinal, immunosuppressive, bacterial, and oncological diseases [9,10]. They are effective and safe drugs, characterized by good pharmacodynamic and pharmacokinetic characteristics, including resistance to digestive enzymes, affinity and selectivity for a molecular target, and favorable absorption (A), distribution (D), metabolism (M), excretion (E), and tolerable toxicity (T) (ADMET). These compounds, however, are not of natural origin. Thus, their use may be associated with the occurrence of undesirable health effects. Therefore, natural foods can provide bioactive compounds while eliminating the negative effects of synthetic compounds. It should also be noted that the bioactive effect of peptides isolated from food cannot be the only method of treating type II diabetes. Meat and meat products can, however, be used preventively or as a method to support the treatment of lifestyle-related diseases, including diabetes. Moreover, dietary proteins and protein hydrolysates have GRAS status in the United States and are allowed in most countries. Amino acids and peptides are components of dietary supplements [11,12]. 

Research on the effects of bioactive proteins and peptides in food is difficult due to the technical limitations of in vivo studies and the natural tendency to hydrolyze proteins in the human gastrointestinal tract. A solution to this may be in silico analysis, at least for the identification and prediction of biological activity of peptides present in food. Previous research on the identification of a bioactive food-derived peptide has found that not only resistance to digestive enzymes but also ADMET is an important parameter in evaluating potential food ingredients for improving consumer health [13]. Thus, in research on peptides as part of a functional food of a pro-health nature, the peptides must meet the same criteria (ADMET) as those for commonly available drugs in order to be able to demonstrate their bioactive effect in vivo. Therefore, in the present study, apart from the identification of peptides released during in vitro hydrolysis from pork loins (after 28, 90, 180, 270, and 360 days), the properties of peptides obtained using bioinformatics tools (in silico analysis: BIOPEP-UWM, SwissTargetPrediction, and ADMETlab) were also assessed in terms of their ability to act as DPP-IV inhibitors after oral administration with the same criteria as those used for drugs.

## 2. Materials and Methods

### 2.1. Dry-Cured Loin Preparation

The study was performed using dry-cured meat products, which were prepared from loins (*M. longissimus thoracis*) with an average weight of 2.00 ± 0.50 kg excised at 24 h postmortem from Polish Large White purebred fatteners with a body weight of approximately 125 kg at slaughter (Lublin, Poland).

The final product was prepared as follows: curing (by surface massage with a mixture of 20 g of sea salt, 9.7 g of curing salt, and 0.3 g of NaNO_3_ per kg of tender loin, 24 h, 4 °C), post-curing stage (in a laboratory for aging, 16 ± 1 °C, relative humidity from 75% to 80% for 14 days), and general maturation (pork loin pieces were vacuum packed in PA/PE bags (80 μm thick) (Wispak, Lublin, Poland) and aged at 4 ± 1 °C for 360 days). Four independent experimental trials were conducted. For the study, samples were selected for analysis at five different time points of processing: after 28, 90, 180, 270, and 360 days of aging.

### 2.2. Muscle Protein Extraction and Hydrolysis

WSF and SSF of meat muscle proteins were extracted based on their solubility criteria according to Molina and Toldrá [14] and Fadda et al. [15]. The WSF was obtained by homogenizing meat tissue in cold water at the ratio of 1:10 for 2 min (T25 Basic ULTRA-TURRAX; IKA, Staufen, Germany) and then centrifuging the extracts (10,000× *g*, 4 °C for 10 min). The obtained supernatant was collected, and the resulting pellet from the WSF extract was used to obtain the SSF fraction. For this purpose, the pellet was re-suspended in 0.6 M NaCl in 0.1 M phosphate buffer (pH 6.2) in the ratio of 1: 6 and homogenized for 2 min. The resulting homogenate was deaerated prior to extraction for 18 h at 4 °C, and the extracts were centrifuged (10,000× *g*, 4 °C for 10 min). After each centrifugation step, the supernatant was filtered through a Whatman Filter Paper No. 1 and used for further analysis. Protein concentration of both fractions was determined by the Bradford method [16] with bovine serum albumin (BSA) as the standard. Muscle protein fractions (WSF and SSF) were subjected to in vitro hydrolysis by pepsin and pancreatin [17]. The pH of the extracts (50 mL) was adjusted to 2.0 with 1 M HCl before hydrolysis. Later, pepsin was added in an enzyme-to-substrate ratio of 1:100. After a gastric digestion step (2 h at 37 °C in dark and with constant stirring), the enzyme was deactivated by adjusting the pH to 7.0 with 1 M NaOH. The remaining pancreatin was then added at the enzyme-to-substrate ratio of 1:50. After an intestinal digestion step (3 h at 37 °C in dark and with constant stirring), the enzyme activity was terminated by heating at 95 °C for 10 min and allowed to cool. The obtained reaction mixture was collected and subjected to further analysis.

### 2.3. Peptide Fractions and Spectrometric Identification

Peptides were isolated from hydrolysates by dilution (1:4, phosphate buffered saline at pH 7.4) and ultrafiltration (molecular weight cutoff 7 kDa; Spectra/Por^®^). Next, the obtained fractions were concentrated in the evaporator, and the peptides were dissolved in 0.01 M HCl. The peptides were then analyzed by liquid chromatography coupled with tandem electrospray mass spectrometry (LC–MS/MS). The samples were concentrated and desalted on an RP-C18 pre-column (Waters Corp., Milford, MA, USA), and peptide separation was achieved on an RP-C18 nano-Ultra Performance column (Waters) by using a 180 min linear acetonitrile gradient (0–35%) at the flow rate of 250 nL·min^−1^. The outlet of the column was directly connected to a mass spectrometer (Orbitrap Velos, Thermo Fisher Scientific Inc., Waltham, MA, USA). The raw data files were preprocessed using Mascot Distiller software (version 2.4.2.0, Matrix Science Inc., Boston, MA, USA). The obtained peptide masses and their fragmentation pattern were compared with the protein sequence database (UniProtKB, www.uniprot.org, accessed February 2021) by using the Mascot search engine (Mascot Daemon v. 2.4.0, Mascot Server v.2.4.1, Matrix Science, London, UK). The “mammals” option was chosen as the taxonomy constraint parameter. The following search parameters were applied: enzyme specificity, none; peptide mass tolerance, 5 ppm; fragment mass tolerance, 0.01 Da. The protein mass was left unrestricted, and mass values were monoisotopic with a maximum of two missed cleavages allowed. Methylthiolation, oxidation, and carbamidomethylation were set as fixed and variable modifications. The sequences of peptides from unknown original proteins were not listed. Peptide identification was performed using the Mascot search engine (Matrix Science) with a probability-based algorithm. An expected value threshold of 0.05 was used for the analysis (all peptide identifications had less than 0.05% chance of being a random match) [18].

### 2.4. Peptide Characterization and DPP IV Inhibitory Activity Determination—Chemometric Analysis

#### 2.4.1. In Silico Bioactivity Prediction

First, a potential biological activity profile was determined to identify sequences with high potential to inhibit the action of DPP-IV. The profile of potential biological activity of peptides and the frequency of bioactive fragment occurrence in a protein sequence (parameter *A*) were determined using the tools available in the BIOPEP-UWM database [19]. Parameter *A* is available in tab “Calculations,” and it is defined by the following formula:

(1) $$A\geq \frac{a}{N}$$

where: a—the number of fragments with given activity in a protein sequence and *N*—the number of amino acid residues of a protein.

#### 2.4.2. In Silico Peptide–Protein Interaction Prediction

The STRING database [20] was used to determine possible interactions between proteins that were identified as a good source of stable DPP-IV inhibitory peptides. A functional interaction network linking the proteins identified from the peptides released during aging was presented graphically, and Sus scrofa was chosen as the detection parameter in the analysis.

#### 2.4.3. SwissTargetPrediction

Properly identifying and validating biomolecule–target interactions is the first step in the drug-like molecule discovery process. For this purpose, SwissTargetPrediction web tools were used [21]. SwissTargetPrediction is a web server used to infer the targets of bioactive small molecules based on the combination of 2D and 3D similarity values with peptides as ligands. The obtained results were limited to presenting the probability of interaction of the selected peptides with the DPP-IV molecule.

#### 2.4.4. ADMET Prediction

One of the features of biologically active food-derived peptides is insufficient evidence of their resistance to various enzymes of the metabolic pathway that occur in the human body after ingestion. Moreover, despite many positive features, due to the multifunctional action of biologically active peptides, their negative impact on humans is also possible, such as neurotoxic, anorectic, or opioid effects. Therefore, it is important to consider the additional processes that peptides may undergo after passing through the intestinal walls into the bloodstream. Hence, we assessed the efficacy and potential safety of peptides from dry-cured pork loins by conducting ADMET analysis. In this study, the selected peptide sequences acting as DPP-IV inhibitors were analyzed through an online platform called ADMETlab [22]. ADMET analysis included log Caco-2 permeability and human intestinal absorption (HIA) as adsorption steps; plasma protein binding (PPB) and blood–brain barrier (BBB) penetration as distribution steps; cytochrome P450 (CYP450) 2D6 inhibition prediction as the metabolism step; determination of the half-life index (T_1/2_) as the excretion step; and finally, determination of acute toxicity (LD50) as the toxicity step. Because ADMETlab uses peptide codes, these codes need to be translated into a Simplified Molecular Input Line Entry Specification (SMILES) by BIOPEP-UWM.

## 3. Results and Discussion

### 3.1. Peptide Presentation Based on Spectrometric Analysis

Following the advances in mass spectrometry technology, this technique is being increasingly used to determine various classes of proteins and peptides with a wide range of properties. For example, these methods were used to determine peptide biomarkers, including those helpful in determining the composition of products or their adulteration [23]. As part of this approach, in the present study, potential peptides that were stable during long-term maturation of loins were analyzed spectrometrically and then screened for the presence of potential DPP-IV inhibitory peptide biomarkers in the BIOPEP-UWM knowledge base. Because different properties of meat proteins are related to their solubility in the environment of salt ions, analyses were performed separately for the water-soluble fraction (WSF) and the fraction obtained by extracting proteins in salt solution (salt-soluble fraction [SSF]). Based on the spectrometric analysis, preceded by chromatographic separation, a total of 10,528 sequences of peptide fragments were identified (4912 for WSF and 5616 for SSF). Further analysis, however, used a rigorous approach to detect peptide sequences found in four independent analyses. Subsequently, peptide sequences occurring in at least three of the five periods (to highlight their relatively high stability during aging) were analyzed in detail. The final data are shown in Table 1 and Table 2. More fragments as potential biomarkers were detected for WSF (31 sequences) than for SSF (17 sequences). This trend corresponds to the observations previously reported in the literature wherein WSF was found to be more susceptible to proteolytic changes during long-term aging of dry-cured pork loins [24]. 

The analysis of the aging period showed that the majority of DPP-IV inhibitors were associated with the initial phase (test period 1–3) or the end phase (test period 3–5). This is likely due to progressive proteolysis over time; thus, it is not surprising that some sequences are period-specific. The analysis of the data presented in Table 1 and Table 2 show that the DPP-IV inhibitory peptide was abundant in the product after 90 and 270 days of maturation and after one year of aging (360 days). Previous studies have shown that the biological activity of peptides from raw ripening tender loin is dependent on the duration of the production process and indicated 180 and 270 days, respectively, as the best ripening time for the tender loin in terms of antioxidant activity and ACE-I inhibition by peptides [4,5].

For the identified potential biomarkers of biologically active peptides with DPP-IV inhibitory activity, the frequency of bioactive fragments in the protein sequence (parameter A) was also determined using the “Calculations” tool available in the BIOPEP-UWM database. This parameter is based on the ratio of the number of fragments with given activity in a protein sequence and the number of amino acid residues of the protein. A higher value of parameter A should be considered as a higher probability of exerting a bioactive effect, including interaction of the analyzed sequence with receptors on the DPP-IV molecule. Among all the analyzed sequences, the highest value of parameter A was determined for the WTIAVPGPPHS peptide (A = 0.9091) for WSF and for the FKRPPL peptide (A = 0.8333) for SSF. Because of the high probability of the selected peptides to act as DPP-IV inhibitors, they were selected and subjected to further detailed computational analyses (Section 3.3 and Section 3.4).

### 3.2. Analysis of the Protein Functional Interaction Networks

**Figure 1:**
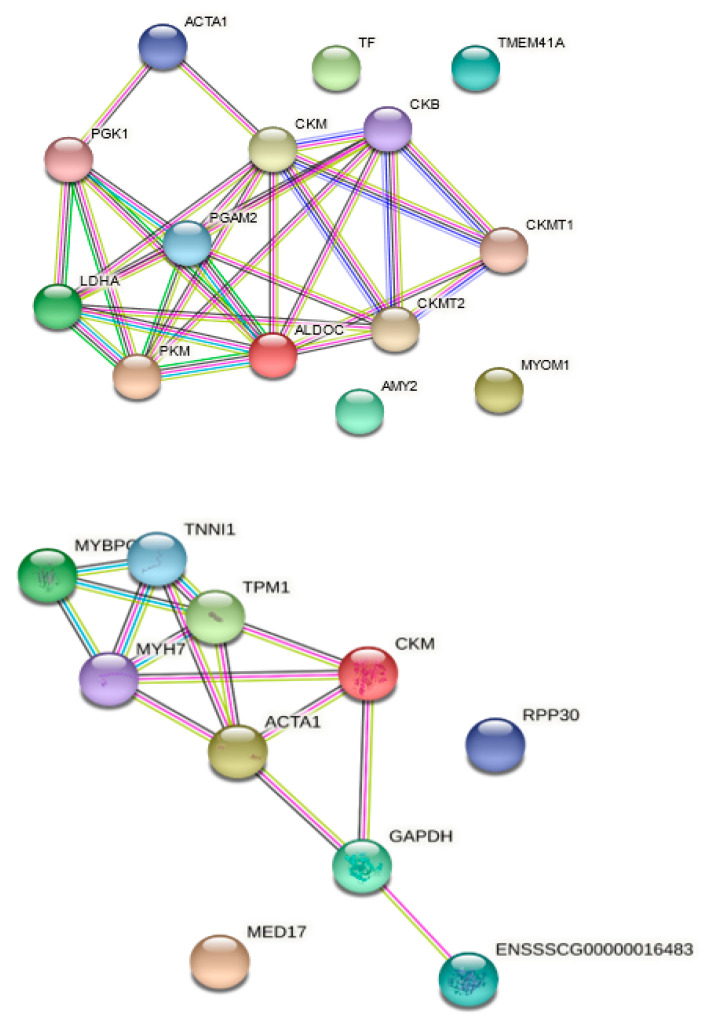
Functional interaction network linking the WSF (above) and SSF (bellow) identified from the peptides released during the aging of dry-cured pork loins (protein names according to UniProtKB).

Pork muscles contain a variety of proteolytic enzymes, especially endo- and exopeptidases, which play an important role in the proteolysis of myofibrillar and sarcoplasmic proteins during the dry curing process. Numerous biological pathways are found to be involved in the physiological, biochemical, and metabolic changes during muscle-to-meat transformation in postmortem muscle [25]. Meat tissue also contains a number of other protein enzymes, such as ATPases, and other enzymes involved in the breakdown of glycogen along with meat oxidoreductases. Their activity may contribute to the pool of peptides released during aging of dry-cured pork loin. The analysis of the obtained peptide-rich extracts by liquid chromatography and tandem mass spectrometry (LC-MS-MS) enabled the identification of proteins as a good source of peptides with high potential for bioactivity toward DPP-IV inhibition. These proteins are presented in the form of a proteome network map, in which their functional relationships are represented by nodes and interactions. This approach, described as an example of functional proteomics, enables the prediction of de novo functional links between the analyzed proteins and provides a detailed understanding of the enzymatic pathways of the proteins of pork muscle tissue during the aging of the meat product. For this purpose, an online database resource search tool for retrieving interacting genes (STRING) was used. STRING is the application that includes hundreds of organisms from bacteria and archaea to humans. Sus scrofa was selected as the target organism to limit the search results. The analyses were performed and reported separately for WSF and SSF (Figure 1).

To the best of our knowledge, no information is currently available on the grouping of meat proteins as parent units for aging-stable bioactive peptides. The STRING database was used to represent the functional network of interactions linking the identified meat proteins. At this stage of the analysis, it was observed that the vast majority of proteins were related to each other in the resulting network (Figure 1). In meat, proteins can be classified according to their muscle function and/or location in the muscle cell. For example, there are structural proteins, the most dominant of which are muscle contraction proteins, i.e., myosin, actin, tropomyosin, and troponin as well as sarcoplasmic proteins involved in metabolic pathways (glycolysis); proteins in organelles such as mitochondria; and finally, collagen as an example of extracellular matrix protein [26]. According to the results of this study, the stability of peptides with DPP-IV inhibitor activity does not depend directly on the protein cascades belonging to different metabolic pathways, and no explicit clustering was established.

The network analysis was carried out using the STRING bioinformatics tools and showed that the main cluster of WSF proteins was associated with proteins responsible for the regulation of muscle contractile activity (e.g., ACTA1, APM3); enzymes playing an important role in energy processes in the body’s cells, mainly glycolysis (CKMT2, PGAM2, PGK1, TPM3, PKM, LDHA, and ALDOC); and others related to the metabolism of polysaccharides (e.g., AMY2). On the other hand, in the field of SSF, the greatest interactions were noted in MYH7, TPM1, or TNN, i.e., in muscle contractile proteins. The observed separation is a characteristic of proteins typically present in pork muscle tissue, i.e., the group of sarcoplasmic (water-soluble) and myofibrillar (salt-soluble) proteins and is consistent with the biochemical functions assigned to them. Among the proteins presented in Figure 1, the proteins MED17 (mediator of the transcriptional subunit 17 of RNA polymerase II) and RPP30 (p30 ribonuclease P/RP subunit) did not interact with other proteins but showed high stability against the time criterion and were found to be an important source of anti-DPP-IV peptides. The obtained results are similar to those reported for dry-cured meat products where the proteomic approach was used to assess the degree of proteolysis during aging [27,28,29,30]; however, the present studies add value by indicating them as a source of stable peptides with DPP-IV inhibitor activity.

### 3.3. Peptides–Enzymes Interaction Prediction

**Figure 2:**
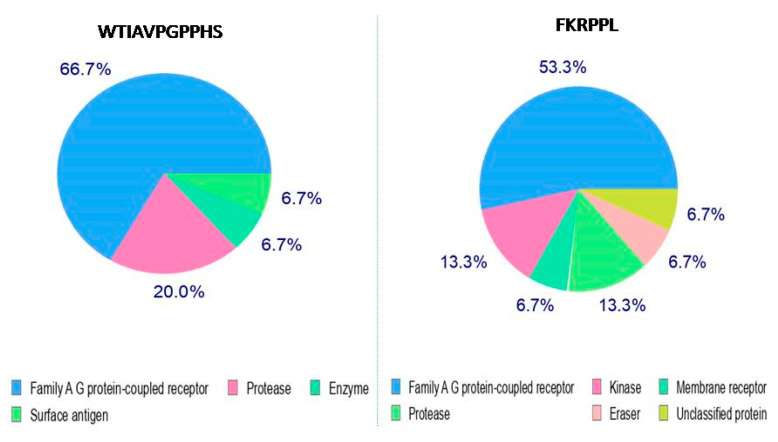
Family of target proteins from the WTIAVPGPPHS and FKRPPL peptides based on the SwissTargetPrediction tool.

Mapping the bioactive targets of small molecules such as peptides is a key step toward discovering the molecular mechanisms underlying their bioactivity and predicting their potential effects [31]. The present study assessed whether the selected (based on parameter A) sequences of the WTIAVPGPPHS peptide and the FKRPPL peptide were able to interact with larger proteins (in this case, the DPP-IV molecule) (Figure 2).

The results of this analysis presenting the classes of proteins (15 most likely proteins) as the potential targets for particular peptides are summarized in Table 3 and Table 4. For this purpose, the SwissTargetPrediction tool was used. This tool is based on the observation that similar bioactive molecules are more likely to have similar target properties. Therefore, the targets of a molecule can be predicted by identifying proteins with known ligands that are very similar to the molecule under study.

It should be noted that the study of peptides as an element of functional food is primarily related to the difficulties resulting from the low availability of peptides administered orally. This occurs because of their inactivation by gastric acid and proteases involved in digestion in the gastrointestinal tract and difficulties in overcoming the intact intestinal barrier, which frequently ends with the breakdown of bioactive peptides into smaller fragments under the action of intestinal enzymes or blood peptidase [32]. Moreover, according to the information available in the literature, the majority of peptides showing biological activity, for example, ACE inhibition, are dipeptides. Furthermore, peptides containing proline at the C-terminus are resistant to gastrointestinal proteolytic enzymes and can be absorbed into the bloodstream in their active form. Thus, peptides with this structure can more easily penetrate the gastrointestinal tract into the bloodstream [33,34]. Therefore, in the subsequent analysis, shorter two- or three-amino-acid sequences of the selected larger peptides were considered. The highest probability value (0.4592) was obtained for the PL peptide as the DPP-IV ligand, followed by PP (0.3095). The above result suggests that it is a promising candidate in this regard. The analyses also showed sequences of peptides with a low probability of interaction with the DPP-IV molecule, i.e., HS, PH, and KR, for which the probability index was 0.00.

### 3.4. ADMET Prediction

Considerations about drug-like biomolecules should begin with the stages of digestion and absorption, which again depend on their inherent bioavailability. In drug development, the pharmacokinetic and pharmacodynamic properties of potential drug candidates are as important as their efficacy, specificity, and safety [35]. This is important because, in pharmacological terms, potential distributed metabolites will act directly on cellular systems, inactive metabolites may inactivate the administered compound and reduce its in vivo activity, and neutral metabolites will be automatically excreted from the kidneys [36]. Assessment of these specific properties, namely ADMET (absorption, distribution, metabolism, excretion, toxicity) for food peptides is therefore essential in their initial stage of identification as a biomolecule. This approach of predicting the drug-likeness of the molecule based on ADMET has recently become a mandatory step in the in silico design of pro-health agents from food [37,38,39,40,41]. In the research process for the identification of bioactive peptides from food, ADMET is an important parameter due to the possible modification of their properties, which are difficult to assess by in vitro or in vivo methods. Therefore, the ADMET analysis is a reliable theoretical tool to evaluate in silico aspects such as absorption or metabolism with excretion. If a compound is rapidly absorbed, well distributed, minimally broken down metabolically, not rapidly cleared, and nontoxic, it is more likely to reach peak blood levels quickly and maintain the desired levels for an extended period of time before being excreted [42]. In the present study, the ADMET prediction of peptides (i.e., GP, PP, VP, IA, WT, AV, HS, PG, TI, RP, PL, RPL, and KR) was performed online using ADMETlab (Table 5).

The first parameter, i.e., the adsorption of peptides after oral administration, is an important parameter for the development of bioactive molecules. To predict peptide absorption, Caco-2 cell models are used as a reliable in vitro model. Caco-2 cells are well-differentiated human colon and rectal gut cell lines of human origin, and the morphological and functional properties of the intestinal epithelial cell barrier in vivo are mapped; therefore, these cells when grown as monolayers are recommended as models to simulate the absorption of compounds from the gastrointestinal tract. However, due to the difficulties in passaging and maintaining the cell lines in vitro, database-based (in silico) analyses such as ADMET can also be performed without the mentioned difficulties. The peptide permeation rate through Caco-2 cells was assessed using the platform for systematic ADMET evaluation, and as the decisive criterion, the optimal result is the log permeability exceeding −5.15 (in accordance with the criterion used by ADMETlab). The predicted permeability of the Caco-2 peptides isolated in this study was satisfactory. Almost all peptides met the adopted criterion, except for HS (−1.721). However, two peptides, namely PP (−5.055) and VP (−5.068), almost reached the cutoff value. According to Table 5, four peptides (WT, RP, RPL, and KR) containing arginine, tryptophan, lysine, or proline had the highest predicted log Caco-2 permeability (greater than −6.0) and thus achieved a high predicted probability of intestinal absorption. Human intestines adsorption (HIA) was used to describe the feasibility parameters of the intestinal absorption of the aforementioned 11 peptides (i.e., DNF, DFH, VWR, GYR, EGF, MDL, GDL, VDF, SGR, and HGR). HIA was positive only for 4 out of the 15 peptides (VP, IA, AV, and PL), and in this case, the presence of either proline, valine, or alanine seemed to be decisive. Among the positively assessed peptides, the probability of HIA was IA > AV > PL > VP, where higher HIA implies that the compound could be better absorbed from the gut after oral administration. It is surprising that out of the 11 peptides, only 4 peptides obtained the beneficial effects of HIA, which is probably due to their differing physicochemical properties [43].

The low-molecular-weight peptides can penetrate the blood–brain barrier (BBB) with slow diffusion through lipids. BBB penetration is a very critical indicator for the evaluation of pharmacological agents, as only CNS-acting compounds can pass through the BBB [44]. These properties also apply to bioactive food ingredients such as peptides, which, due to their multifunctional action, can cause both desired effects and undesirable effects, i.e., anorectic or opioid. The proposed in silico approaches are designed to predict the uptake of compounds entering the brain. The acceptable BBB range for health-promoting compound (including drugs) candidates is −3.0 to 1.2 [45], which was met by all the analyzed peptide fragments. As shown in Table 4, the WTIAVPGPPHS peptide (or rather its smaller fragments) exhibited stronger BBB penetration properties (mean value was 0.840) than the FKRPPL peptide (mean value was 0.43). Among them, the highest coefficient was determined for WT (0.956) and AV (0.932). Plasma Protein Binding (PPB) is another parameter characterizing peptides from dry-cured loin after hydrolysis as biomolecules. Protein binding may increase or decrease the effect of the drug. Although a drug is considered to be clinically significant when the drug protein binding is greater than 80%, the PPB value in this study was in the range of 13.60–45.60%. This value indicates poor plasma protein binding of the compounds. On the other hand, however, agents that are minimally bound to proteins penetrate tissues better than those that are strongly bound but are excreted much faster [46,47]. Therefore, the low PPB value obtained for the peptides in this study should also be considered with other ADMET parameters before final evaluation.

Subsequently, through the small intestine, potential interactions between these peptides and the CYP450 enzymes were assessed based on their metabolic properties. The CYP group of enzymes are the main and most frequently studied phase I drug metabolizing enzymes that mediate the oxidation of various compounds involved in various physiological and pathophysiological processes, including detoxification of xenobiotic compounds. It is estimated that approximately 75% of the drugs available in the market are metabolized by CYP, with the five major CYP isoforms involved in 75–90% of CYP-mediated metabolism [48]. In the present study, CYP4502D6 was examined as a representative of one of the most studied drug metabolizing enzymes. Our studies showed that the analyzed peptides had nonsubstrate/noninhibitory status in relation to the enzyme CYP405 2D6 (except RPL). A CYP450 noninhibitor implies that the molecule will not impede the biotransformation of drugs metabolized by the CYP450 enzyme. On the other hand, substrate molecules can be metabolized by CYP450 [45]. As presented in Table 5, only the RPL peptide has “substrate” status and will therefore be metabolized by CYP 2D6.

The next step, excretion, is determined by the theoretical half-life (T_1/2_) values. As reported by Dong et al. [49], the T_1/2_ ratio should be over 8 h in order to be able to claim that the bioactive molecule is as stable as drugs, and potential drugs are considered to have a short half-life if the T_1/2_ ratio does not exceed 3 h. In the present study, the T_1/2_ for the peptides was moderately short. A calculated half-life of greater than 1 h was observed for the peptide AV (1.229), TI (1.124), and IA (1.114). Moreover, the peptides were characterized by a T_1/2_ value of >0.800. Only three peptides had values around 0.5 (PH (0.588); RP (0.573); and HS (0.550)).

Toxicity as the last measure of ADMET assessment was determined using the LD50 (median lethal dose). This indicator usually represents the acute toxicity of a chemical. This is the dose of the test molecule that kills 50% of the treated animals over a given period. In the comparison of LD50 values, a compound with a lower dose is considered to be more lethal than that with a higher LD50 value [45]. Based on the obtained results (Table 5), the mean LD50 level was 2.336, except for the GP (1904) peptide, which was the most toxic among the ligands tested.

## 4. Conclusions

As demonstrated in this study, integrated global proteomics results and bioinformatics proteome analysis of dry-cured pork loin were combined to provide a comprehensive understanding of peptide DPP-IV inhibitors from meat from a functional point of view. Control of the peptide properties on the basis of the various values obtained in this study indicated that the major drug traits were met in terms of the ADMET profile. Among the selected candidates, AV, IA, and PL had the best ADMET properties, although they did not achieve the highest rates of interference with DPP-IV as the ligand as assessed by SwissTargetPrediction (it should, however, be noted that PL achieved the highest recorded result of 0.4592). The present data increases the potential of these compounds to achieve the desired pharmacokinetic profile and can be used to design compounds with the desired pharmacokinetic profiles. To further ensure the efficacy of the peptide sequences, their synthetic form should be assessed in vitro and in vivo prior to the final evaluation of their antidiabetic activity.

## Figures and Tables

**Table 1:** Peptide sequences obtained from WSF during 12 months of aging of dry-cured pork loins.

No.	Peptide Sequences	Protein Source (UNIPROT ID)	Days					A Parameter	DPP-IV Inhibiting Peptides [Position in Sequences]
			28	90	180	270	360		
1	AGFAGDDAPR	Actin (P68137; ACTA1)	+	−	−	+	+	0.5000	FA [3–4], AP [8–9], AG [1–2] [4–5], GF [2–3]
2	AGLKPGEL	Phosphoglyceratemutase (B5KJG2; PGAM2)	+	+	+	−	−	0.6250	KP [4–5], GL [2–3], AG [1–2], GE [6–7], PG [5–6]
3	ALESPERPF	Phosphoglyceratekinase (F1RPH0; PGK1)	+	+	+	+	+	0.5556	SP [4–5], RP [7–8], AL [1–2], ES [3–4], PF [8–9]
4	DQALKPTKPM	Phosphoglyceratemutase (B5KJG2; PGAM2)	+	−	+	+	+	0.8000	KP [5–6] [8–9], AL [3–4], DQ [1–2], PM [9–10], PT [6–7], QA [2–3], TK [7–8]
5	ELDQALKPTKPM	Phosphoglyceratemutase (Q32KV0; PGAM2)	−	−	+	+	+	0.6667	KP [7–8] [10–11], AL [5–6], DQ [3–4], PM [11–12], PT [8–9], QA [4–5], TK [9–10]
6	GVDNPGHPF	Creatinekinase (Q2HYU1; CKMT2)	+	+	+	−	−	0.8889	HP [7–8], NP [4–5], DN [3–4], GH [6–7], GV [1–2], PF [8–9], PG [5–6], VD [2–3]
7	IFQEVIDLGGEAIK	Alpha-amylase (P00689; AMY2)	−	+	−	+	+	0.4286	EV [4–5], FQ [2–3], GE [10–11], GG [9–10], QE [3–4], VI [5–6]
8	IQLVEEELDRA	Tropomyosin alpha-3 chain (A1XQV4; TPM3)	+	−	−	+	+	0.5455	RA [10–11], DR [9–10], IQ [1–2], LV [3–4], QL [2–3], VE [4–5]
9	KDLFDPIIQDR	Creatine kinase M-type (Q5XLD3; CKM)	+	−	−	+	+	0.5455	DP [5–6], DR [10–11], II [7–8], IQ [8–9], PI [6–7], QD [9–10]
10	KVVDVGSKV	Pyruvate kinase PKM (P11974; PKM)	+	+	−	−	+	0.6667	VV [2–3], KV [1–2] [8–9], SK [7–8], VD [3–4], VG [5–6]
11	LDQALKPTKPM	Phosphoglyceratemutase (B5KJG2; PGAM2)	+	-	+	+	+	0.7273	KP [6–7] [9–10], AL [4–5], DQ [2–3], PM [10–11], PT [7–8], QA [3–4],TK [8–9]
12	LFDKPVSPI	Creatine kinase B-type (Q29594; CKB)	+	+	+	−	−	0.5556	SP [7–8], KP [4–5], PI [8–9], PV [5–6], VS [6–7]
13	LFDKPVSPL	Creatinekinase (Q2HYU1; CKMT2)	+	+	+	+	+	0.5556	SP [7–8], KP [4–5], PI [8–9], PV [5–6], VS [6–7]
14	MSHLGRPDGIPMPD	Phosphoglyceratekinase (F1RPH0; PGK1)	+	+	−	+	−	0.5714	MP [12–13], IP [10–11], RP [6–7], HL [3–4], GI [9–10], PM [11–12], SH [2–3], IPM [10–12]
15	MSHLGRPDGIPMPDK	Phosphoglyceratekinase (F1RPH0; PGK1)	+	+	+	−	+	0.5333	MP [12–13], IP [10–11], RP [6–7], HL [3–4], GI [9–10], PM [11–12], SH [2–3], IPM [10–12]
16	NLHPELGTDADKEH	L-lactate dehydrogenase A chain (P00339; LDHA)	+	−	−	+	+	0.5000	HP [3–4], AD [10–11], EH [13–14], KE [12–13], LH [2–3], NL [1–2], TD [8–9]
17	PEILPDGDHD	Fructose-bisphosphate aldolase (F1RJ25; ALDOC)	+	+	+	+	+	0.4000	LP [4–5], EI [2–3], HD [9–10], IL [3–4]
18	PEILPDGDHDL	Fructose-bisphosphate aldolase (F1RJ25; ALDOC)	+	+	+	+	+	0.3636	LP [4–5], EI [2–3], HD [9–10], IL [3–4]
19	PEILPDGDHDLK	Fructose-bisphosphate aldolase (F1RJ25; ALDOC)	+	+	+	+	+	0.3333	LP [4–5], EI [2–3], HD [9–10], IL [3–4]
20	SAPILNIPI	Transmembrane protein 41A (I3LPS6; TMEM41A)	+	+	+	−	−	0.7778	IPI [7–9], AP [2–3], IP [7–8], IL [4–5], LN [5–6], PI [3–4] [8–9]
21	SAPILNIPIV	Transmembrane protein 41A (I3LPS6; TMEM41A)	+	+	+	−	+	0.7778	IPI [7–9], AP [2–3], IP [7–8], IL [4–5], LN [5–6], PI [3–4] [8–9]
22	SFDIPPPPMD	Phosphoglyceratemutase (Q32KV0; PGAM2)	−	+	+	+	+	0.7000	PPPP [5–8], PP [5–6] [6–7] [7–8], IP [4–5], PM [8–9], SF [1–2]
23	SFDIPPPPMDEK	Phosphoglyceratemutase (Q32KV0; PGAM2)	+	−	+	+	+	0.6667	PPPP [5–8], PP [5–6] [6–7] [7–8], IP [4–5], EK [11–12], PM [8–9], SF [1–2]
24	SISNSAEDPFIAIH	Alpha-amylase (F1S574; AMY2)	-	+	−	+	+	0.4286	IA [11–12], AE [6–7], DP [8–9], IH [13–14], PF [9–10], SI [1–2]
25	VDLPAVSEK	Multifunctional fusion protein (F1SHL9; PKM)	+	−	−	+	+	0.6667	PA [4–5], LP [3–4], EK [8–9], AV [5–6], VD [1–2], VS [6–7]
26	VGVNLPK	Phosphoglyceratekinase (F1RPH0; PGK1)	+	+	+	+	−	0.8571	LP [5–6], GV [2–3], NL [4–5], PK [6–7], VG [1–2], VN [3–4]
27	VPAPVEIPVTPPTLVSGLK	Titin (Q8WZ42; TTN)	+	-	+	−	+	0.8421	PP [11–12], AP [3–4], PA [2–3], VP [1–2], IP [7–8], TP [10–11], GL [17–18], EI [6–7], LV [14–15], PT [12–13], PV [4–5] [8–9], TL [13–14], VE [5–6], VS [15–16], VT [9–10]
28	WILGEHGDSSVPV	L-lactate dehydrogenase A chain (P00339; LDHA)	+	+	−	−	+	0.5385	VP [11–12], WI [1–2], EH [5–6], GE [4–5], IL [2–3], PV [12–13], SV [10–11]
29	WTIAVPGPPH	Myomesin 1 (F1SM75; MYOM1)	+	−	+	+	+	0.9000	GP [7–8], PP [8–9], VP [5–6], IA [3–4]WT [1–2], AV [4–5], PG [6–7], PH [9–10], TI [2–3]
30	WTIAVPGPPHS	Myomesin 1 (F1SM75; MYOM1)	−	−	+	+	+	0.9091	GP [7–8], PP [8–9], VP [5–6], IA [3–4], WT [1–2], AV [4–5], HS [10–11], PG [6–7], PH [9–10], TI [2–3]
31	YDQLPEPR	Serotransferrin (P09571; TF)	−	+	+	−	+	0.6250	LP [4–5], EP [6–7], DQ [2–3], QL [3–4], YD [1–2]

**Table 2:** Peptide sequences obtained from SSF during 12 months of aging of dry-cured pork loins.

No.	Peptide Sequences	Protein Source (UNIPROT ID)	Days					A Parameter	DPP-IV Inhibiting Peptides [Position in Sequences]
			28	90	180	270	360		
1	AGFAGDDAPR	Actin, alpha skeletal muscle (P68137; ACTA1)	+	−	+	+	+	0.5000	FA [3–4], AP [8–9], AG [1–2] [4–5], GF [2–3]
2	FDKPVSPL	Creatine kinase M-type (Q5XLD3; CKM)	+	+	+	−	−	0.6250	SP [6–7], KP [3–4], PL [7–8], PV [4–5], VS [5–6]
3	FKRPLP	Mediator of RNA polymerase II transcription subunit 17 (F1STK7; MED17)	+	+	+	−	−	0.6667	LP [5–6], RP [3–4], PL [4–5], KR [2–3]
4	FKRPPI	ribonuclease P/MRP subunit p30 (F1SCX5; RPP30)	+	+	+	−	−	0.6667	PP [4–5], RP [3–4], KR [2–3], PI [5–6]
5	FKRPPL	Troponin I (I3LB76; TTI1)	+	+	+	−	−	0.8333	PP [4–5], RP [3–4], PL [5–6], PRL [4–6], KR [2–3]
6	FRVPTPNVSV	Glyceraldehyde-3-phosphate dehydrogenase (P00355; GAPDH)	+	+	−	+	−	0.8000	VP [3–4], TP [5–6], FR [1–2], NV [7–8], PN [6–7], PT [4–5], SV [9–10], VS [8–9]
7	IIAPPER	Actin, alpha skeletal muscle (P68137; ACTA1)	+	+	+	−	−	0.7143	PP [4–5], AP [3–4], IA [2–3], IIAP [1–4], II [1–2]
8	KDLFDPIIQD	Creatine kinase M-type (Q5XLD3; CKM)	+	+	−	+	−	0.5000	DP [5–6], II [7–8], IQ [8–9], PI [6–7], QD [9–10]
9	LGEHNIDVLEGNEQFINAAK	Trypsin (P00761)	+	+	−	+	−	0.4500	AA [18–19], EG [10–11], EH [3–4], GE [2–3], IN [16–17], NA [17–18], NE [12–13], QF [14–15], VL [8–9]
10	LTEAPLNPK	Actin, alpha skeletal muscle (P68137; ACTA1)	+	+	−	+	−	0.7778	AP [4–5], NP. [7–8], PL [5–6], LN [6–7], LT [1–2], PK [8–9], TE [2–3]
11	LVIIESDLER	Tropomyosin alpha-1 chain (P42639; TPM1)	+	−	−	+	+	0.4000	ES [5–6], II [3–4], LV [1–2], VI [2–3]
12	PEILPDGDHDL	Fructose-bisphosphate aldolase (P09972; ALDOC)	+	+	−	+	−	0.3636	LP [4–5], EI [2–3], HD [9–10], IL [3–4]
13	PEILPDGDHDLK	Fructose-bisphosphate aldolase (P09972; ALDOC)	+	+	−	+	−	0.3333	LP [4–5], EI [2–3], HD [9–10], IL [3–4]
14	PVTIPDKPNSE	Calsequestrin-1 (P31415; CASQ)	−	+	+	+	−	0.5455	IP [4–5], KP [7–8], PN [8–9], PV [1–2], TI [3–4], VT [2–3]
15	PVTIPDKPNSEE	Calsequestrin-1 (P31415; CASQ)	−	+	+	+	−	0.5000	IP [4–5], KP [7–8], PN [8–9], PV [1–2], TI [3–4], VT [2–3]
16	RVVDVPDPPEA	Myosin binding protein C2 (F1RH19; MYBPC2)	+	+	+	-	−	0.4545	PP [8–9], VP [5–6], VV [2–3], DP [7–8], VD [3–4]
17	VDDLEGSLEQEK	Myosin-7 (P79293; MYH7)	+	+	−	+	−	0.4167	EK [11–12], SL [7–8], EG [5–6], QE [10–11], VD [1–2]

**Table 3:** The result of predicting the likelihood of bioactive small peptides acting as DPP-IV ligands.

WTIAVPGPPHS		
Sequences	SMILES Code	Probability ^1^
GP	NCC(=O)N1[C@@]([H])(CCC1)C(=O)O	0.1334
PP	N1[C@]([H])(CCC1)C(=O)N1[C@]([H])(CCC1)C(=O)O	0.3095
VP	N[C@@]([H])(C(C)C)C(=O)N1[C@@]([H])(CCC1)C(=O)O	0.1858
IA	N[C@@]([H])([C@]([H])(CC)C)C(=O)N[C@@]([H])(C)C(=O)O	0.0619
WT	N[C@@H](Cc1c[nH]c2c1cccc2)C(=O)N[C@@]([H])([C@]([H])(O)C)C(=O)O	0.1273
AV	N[C@]([H])(C)C(=O)N[C@]([H])(C(C)C)C(=O)O	0.1684
HS	N[C@H](Cc1c[nH]cn1)C(=O)N[C@]([H])(CO)C(=O)O	0.0000
PG	N1[C@@]([H])(CCC1)C(=O)NCC(=O)O	0.1501
PH	N1[C@@]([H])(CCC1)C(=O)N[C@@H](Cc1c[nH]cn1)C(=O)O	0.0000
TI	N[C@@]([H])([C@]([H])(O)C)C(=O)N[C@@]([H])([C@]([H])(CC)C)C(=O)O	0.0864

^1^—Probability of peptide interaction with DPP-IV molecule.

**Table 4:** The result of predicting the likelihood of bioactive small peptides acting as DPP-IV ligands.

FKRPPL		
Sequences	SMILES Code	Probability ^1^
PP	N1[C@]([H])(CCC1)C(=O)N1[C@]([H])(CCC1)C(=O)O	0.3095
RP	N[C@]([H])(CCCNC(=N)N)C(=O)N1[C@]([H])(CCC1)C(=O)O	0.1120
PL	N1[C@]([H])(CCC1)C(=O)N[C@]([H])(CC(C)C)C(=O)O	0.4592
RPL	N[C@@]([H])(CCCNC(=N)N)C(=O)N1[C@@]([H])(CCC1)C(=O)N[C@@]([H])(CC(C)C)C(=O)O	0.1213
KR	N[C@]([H])(CCCCN)C(=O)N[C@]([H])(CCCNC(=N)N)C(=O)O	0.0000

^1^—Probability of peptide interaction with DPP-IV molecule.

**Table 5:** ADMET profile of the test ligand acting as DPP-IV inhibitors.

Sequences		A		D			M	E	T
		Caco-2 Permeability ^1^	HIA ^2^	PPB ^3^	BBB ^4^	VD ^5^	Cyp450 2D6 ^6^	T_1/2_ ^7^	LD_50_ ^8^
WTIAVPGPPHS	GP	−5.144	0	13.60	0.629	−0.554	0	0.990	1.904
	PP	−5.055	0	25.48	0.716	−0.444	0	0.845	2.423
	VP	−5.068	1 (0.504)	38.18	0.624	−0.448	0	0.924	2.423
	IA	−5.522	1 (0.616)	23.65	0.864	−0.505	0	1.114	2.158
	WT	−6.061	0	53.96	0.956	−0.752	0	0.715	2.211
	AV	−5.540	1 (0.553)	28.01	0.932	−0.596	0	1.229	2.058
	HS	−1.721	0	13.87	0.909	−0.765	0	0.55	2.401
	PG	−5.372	0	15.65	0.899	−0.042	0	0.989	2.020
	PH	−5.863	0	20.78	0.910	−0.510	0	0.588	2.535
	TI	−5.786	0	35.24	0.749	−0.545	0	1.124	2.371
FKRPPL	PP	−5.055	0	25.48	0.714	−0.444	0	0.845	2.432
	RP	−6.200	0	25.84	0.251	−0.604	0	0.573	2.450
	PL	−5.376	1 (0.510)	45.60	0.830	−0.487	0	0.876	2.382
	RPL	−6.248	0	33.58	0.256	−0.840	1 (substrate)	0.723	2.514
	KR	−6.280	0	32.87	0.523	−0.759	0	0.896	2.328

^1^—[Expressed in cm × s^−1^] Optimal: higher than −5.15 Log unit; ^2^—Human Intestinal Absorption, criteria: 0: HIA−, 1: HIA+; ^3^—Plasma Protein Binding [%], optimal: <90%, significant with drugs that are highly protein-bound and have a low therapeutic index; ^4^—Blood–Brain Barrier (BBB), range: BB ratio ≥ 0.1: BBB+, BB ratio < 0.1; ^5^—Value Distribution [L × kg^−1^], optimal: 0.04–20; ^6^—Cyp 450 inhibitor or substrate, criteria: 0: non-inhibitor/substrate, category 1: inhibitor/substrate; ^7^—Half Life, criteria: >8 h: high, from 3 h to 8 h: moderate, <3 h: low; ^8^—LD_50_ of acute toxicity [−log mol/kg].

## Data Availability

Data available on request from the authors.
